# An Open-Label Pilot Study Testing the Feasibility of Assessing Total Symptom Burden in Trials of Cannabinoid Medications in Palliative Care

**DOI:** 10.1089/jpm.2019.0540

**Published:** 2020-05-04

**Authors:** Phillip D. Good, Ristan M. Greer, Georgina E. Huggett, Janet R. Hardy

**Affiliations:** ^1^ Department of Palliative and Supportive Care, Mater Misericordiae, Ltd., Brisbane, Queensland, Australia.; ^2^ Mater Research University of Queensland, South Brisbane, Queensland, Australia.; ^3^ Department of Palliative Care, St. Vincent's Private Hospital, Brisbane, Queensland, Australia.

**Keywords:** cancer, cannabidiol, cannabis, palliative care, symptom control, tetrahydrocannabinol

## Abstract

***Background:*** There is considerable interest in the use of cannabinoids for symptom control in palliative care, but there is little high-quality evidence to guide clinical practice.

***Objectives:*** Assess the feasibility of using global symptom burden measures to assess response to medicinal cannabis, to determine median tolerated doses of cannabidiol (CBD) and tetrahydrocannabinol (THC), and to document adverse events (AEs).

***Design:*** Prospective two-arm open-label pilot trial of escalating doses of CBD and THC oil.

***Setting/Subjects:*** Participants had advanced cancer and cancer-related symptoms in a palliative and supportive care service in an Australian cancer center.

***Measurements:*** The main outcome measures were the number of participants screened and randomized over the time frame, the number of participants completing days 14 and 28 and providing total symptom distress scores (TSDSs) (measured using the Edmonton Symptom Assessment Scale), and the change from baseline of the TSDS at day 14.

***Results:*** Of the 21 participants enrolled (CBD, *n* = 16; THC, *n* = 5), 18 (86%) completed the primary outcome measure at day 14 and 8 completed at day 28. The median maximum tolerated doses were CBD, 300 mg/day (range 100–600 mg); THC, 10 mg/day (range 5–30 mg). Nine of 21 patients (43%) met the definition of response (≥6 point reduction in TSDS). Drowsiness was the most common AE.

***Conclusions:*** Trials of medicinal cannabis in advanced cancer patients undergoing palliative care are feasible. The doses of THC and CBD used in this study were generally well tolerated and the outcome measure of total symptom distress is promising as a measure of overall symptom benefit. Trial registration: ACTRN12618001205224.

## Introduction

Despite recent advances in medicine care, some patients with advanced cancer still experience substantial symptom distress.^1^ To improve symptom control and quality of life (QoL), palliative care aims to provide a holistic individualized approach to medical care. Some symptoms can be difficult to control and there is a need for more effective medications to assist with symptom management.

Medicinal cannabis may have potential to play an important role in symptom control. Cannabis contains ∼500 bioactive compounds, including ∼70 different cannabinoids.^2^ The predominant cannabinoids include delta-9-tetrahydrocannabinol (THC) and cannabidiol (CBD). Although there is considerable interest in the use of cannabinoids for symptom control in palliative care, particularly among consumers, there is little high-quality evidence to guide clinical practice.^3^ Despite the very strong impression of benefit from the lay community, reviews of benefit in the palliative care population have raised many unknowns, for example, what is the ideal product, dose, formulation, and clinical areas of benefit.

As studies of medicinal cannabis that have assessed isolated symptoms in cancer patents have been largely negative, for example, in pain and anorexia,^4,5^ this pilot study was designed to determine the feasibility of assessing symptom burden as a whole, aiming to capture the perceived improvement in general well-being reported anecdotally by many patients who have used cannabis.^6,7^ Symptom burden was measured by using the Edmonton Symptom Assessment Scale (ESAS).^8^

In this feasibility study, both CBD and THC were piloted as single agents before definitive randomized placebo controlled trials of various medicinal cannabis products. Secondary outcomes included dose tolerance, efficacy, patient-perceived benefit, and adverse events (AEs).

## Methods

This is a prospective two-arm open label trial of escalating doses of CBD and THC oil (ACTRN12618001220257). Limited supplies of both approved CBD and THC from companies prepared to support cannabis research restricted number of participants. The choice of study drug was at the discretion of the patient and investigator based on factors including patient preference, product availability, and government regulations prohibiting driving on THC. The study was approved by the Mater Human Research Ethics Committee (HREC/18/MHS/83).

### Population

Patients with advanced cancer and cancer-related symptoms were recruited from the palliative and supportive care service within Mater Health Services in Brisbane. All participants were receiving standard palliative care,^9^ and their current medications were continued and modified according to clinical need. Participants were reviewed each week in an outpatient clinic, assessed for response and AEs, and provided with a new medication supply if appropriate.

### Inclusion criteria

Patients aged >25 years with advanced cancer (metastatic or locally advanced solid tumors or advanced hematological malignancies) who had been referred or known to the palliative care team, who had an ESAS total symptom distress score (TSDS) ≥10 and at least one individual ESAS score ≥3, a performance status AKPS (Australia-modified Karnofsky Scale score) of ≥30 or above, and agreed to use no other cannabis-based product/s for the duration of the trial were eligible.

### Exclusion criteria

Patients were excluded if they had a history of hypersensitivity to any cannabinoid, unstable untreated cardiovascular disease, severe hepatic or renal impairment, a history of psychiatric disorders, cognitive impairment, known substance use disorder, or a history suggesting that drug diversion may be a risk. Patients could not have participated in a trial of a new clinical entity within the past 28 days, nor had treatment with a new specific anticancer agent within the previous 21 days or radiation therapy within 7 days.

### Medications

Patients were allocated to either of two arms: Arm 1—CBD (CBD 100 mg/mL) oral oily solution—dose range 50 to 600 mg/day, Arm 2—THC (Delta-9-THC 10 mg/mL) oral oily solution—dose range 2.5 to 30 mg/day. The good manufacturing practice approved products GD Cann-C© and Cann-T© were supplied by GD Pharma who had no role in the design of the trial nor interpretation of results. Dose titration was monitored by regular consultation between participant and trial clinicians. Participants were advised to increase their dose every two days according to a set schedule ( Supplementary Appendix 1) until they were satisfied with their symptom improvement or they experienced unacceptable side effects. They were then advised to remain on that dose until the primary outcome point (14 days) and were encouraged to remain on the cannabinoid preparation for continuing assessment of efficacy and AEs for a total of 28 days. Post trial, participants were prescribed medicinal cannabinoid preparations for on-going use if they chose to continue.

### Study outcomes

The primary outcome measure was feasibility as measured by the number of participants screened, number and reason of screen fails, number of participants completing 14 and 28 days, and ability to complete TSDS measurements over time.

Symptom burden was measured by using the ESAS TSDS.^8^ Nine symptoms are scored on a numerical rating scale from 0 to 10 (0 = not a problem, 10 = worst possible). Symptom burden can be represented by the physical scores (sum of pain, fatigue, nausea, drowsiness, appetite, and dyspnea), emotional scores (sum of depression and anxiety), and the well-being score. TSDS is the sum of the physical, emotional scores, and the well-being score. It ranges from 0 to 90, with a higher score representing higher symptom burden. An improvement of 5.7 on the TSDS has been shown to be the minimal clinical important difference.^8^

Secondary outcomes were patient-determined effective doses of THC and CBD (defined as the dose that achieved symptom relief with acceptable side effects), individual, total, physical, and emotional ESAS scores on medical review days (days 7, 14, 21, and 28). Patient and clinician impression of benefit was assessed using patient and clinician global impression of change scales (PGIC and CGIC),^10,11^ along with a depression, anxiety, and stress scale (DASS-21^12^) and QoL (EORTC QLQ-C15-PAL^13^).

AEs were assessed using the National Cancer Institute (NCI) common toxicity criteria.^14^ The known common AEs associated with cannabinoids were specifically addressed at each time point, namely neurological (confusion and somnolence), psychiatric (personality change, paranoia, anxiety, mood changes, and psychosis), cardiovascular (hypertension and tachycardia), systemic (sweating), and gastrointestinal (nausea, vomiting, and abdominal pain) events.

### Analysis and sample size

Descriptive analyses and frequency distributions were generated from participants' demographic and clinical characteristics. Unless otherwise indicated, summary statistics are reported as mean, standard deviation (SD) or mean, standard error for normally distributed data, and as median, interquartile range, or median and range for non-normally distributed data. In preparation for an adequately powered randomized controlled trial, we defined response as a ≥6 point reduction in TSDS at day 14.^8^ TSDS and components of ESAS at day 14 were compared with baseline using a paired *t* test. Other comparisons were made using a paired *t* test for normally distributed data and Wilcoxon's rank sum test for non-normally distributed data. Median survival was estimated using the Kaplan–Meier product limit method. Statistical significance was set at *p* ≤ 0.05. Participants were constituted of a convenience sample. Sample size in this study was limited by availability of the medications supplied.

Study data were entered into a custom-built REDCap database (https://www.project-redcap.org) and analyzed using Stata 13 StataCorp. 2013. Stata Statistical Software: Release 13. College Station, TX: StataCorp LP).

## Results

### Feasibility

**FIG. 1.**
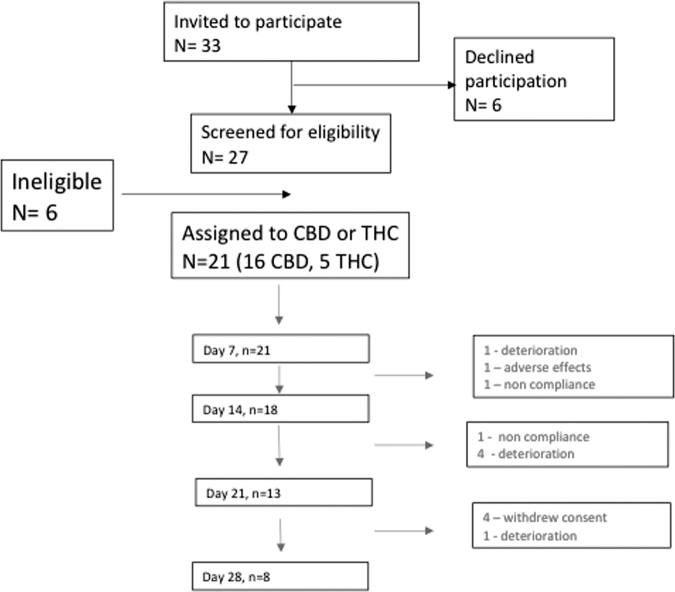
Consort diagram.

Patient flow is shown in Figure 1. Thirty-three people were approached to participate in the study, 27 were screened for eligibility and 21 subjects consented to trial either CBD (*n* = 16) or THC (*n* = 5) for a four-month time period. Eighteen participants completed the primary outcome measure (TSDSs) on day 14 (3 withdrawals, 1 on THC) and a further 10 participants (62% of total, 4 on THC) withdrew by day 28. Reasons for withdrawal were noncompliance (2/21), unacceptable side effects (1/21, drowsiness), and clinical deterioration (6/21). Four participants withdrew consent. Three participants requested ongoing medicinal cannabis poststudy.

### Participant characteristics

The baseline characteristics of participants are given in Table 1. The majority of participants were female (14/21). Most participants had breast cancer as their primary diagnosis. Four of 21 participants tested positive for THC on the urine cannabis pretrial screen (Table 1). All but one participant (20/21) were on an opioid medication at some stage during the study, 8/21 were taking benzodiazepines, and 9/21 were on corticosteroids. The average number of medications per participant was seven. All were receiving standard care from the palliative care team. The median performance status at baseline was 70/100 (range 50–90). The median survival from start of study was 143 days (95% CI 57 days—not estimable) at the census point (4 months after completion of the last participant); six participants died within 1 month of study completion.

**Table 1.** Baseline Characteristics (*n* = 21)

Sex, male/female %	33.3/66.7
Age, mean (SD)	57.5 (12.4) years
OME (median, range)	140 mg (0–800 mg)
THC urine test positive, *n* (%)	4 (19)
Drug allocation, CBD/THC	16/5
AKPS (median, range)	70 (50–90)
TSDS (mean, SD, range)	41.1 (16.52, 14–64)
Cancer, *n* (%)	Breast 7 (33)
	Prostate 4 (19)
	Colorectal 3 (14)
	Gynecological 2 (10)
	Pancreas 2 (10)
	Hematological 1 (5)
	Bone/soft tissue 1 (5)
	Unknown primary 1 (5)
Prior cancer treatment, *n* (%)	Chemotherapy 16 (76)
	Immunotherapy 3 (14)
	Hormone therapy 5 (24)
	RT 15 (71)
	Surgery 13 (62)
Ongoing cancer treatment, *n* (%)	12 (57)
Median survival (from start of study)	143 days (95% CI 57 days—not estimable)

CBD, cannabidiol; OME, oral morphine equivalent; SD, standard deviation; THC, tetrahydrocannabinol; TSDS, total symptom distress score.

### Dose tolerance

The median maximum tolerated dose of CBD was 300 mg/day (range 100–600 mg) whereas the median maximum tolerated dose of THC was 10 mg/day (range 5–30 mg).

### Response

**FIG. 2.**
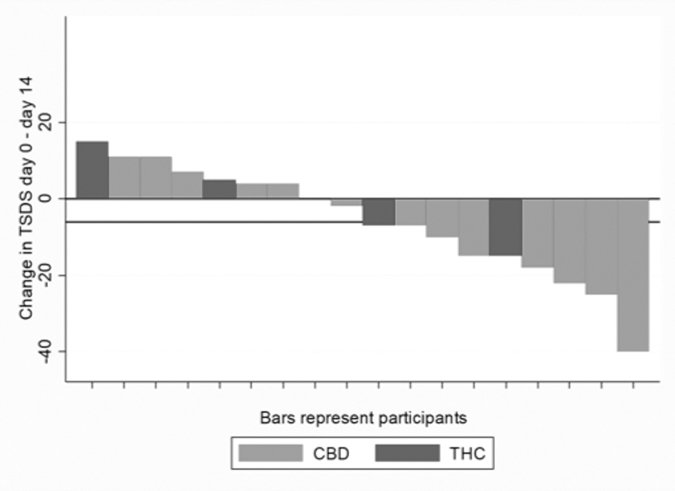
Waterfall plot of response per patient (*n* = 18) at day 14. The lower horizontal line represents a change in TSDS of −6, the defined primary endpoint of the study. TSDS, total symptom distress score.

Nine of 21 patients (43%) met the definition of response (>6 point reduction in TSDS). One participant on CBD had a dramatic improvement in TSDS (−40 points) and seven reported worsening symptom scores (Fig. 2).

Across all patients, there was no significant change in ESAS TSDS from baseline to day 14 (mean [SD] change = −5.8 [14.7], *n* = 18, *p* = 0.11). There was no improvement in the physical subscale of ESAS (mean [SD] change = −2.6 [8.7], *n* = 18, *p* = 0.23) or well-being score (mean [SD] change = −0.3 [2.6], *n* = 18, *p* = 0.65). There was a significant improvement on the emotional subscale (mean [SD] change = −2.9 [4.6], *n* = 18, *p* = 0.01).

The individual symptoms showing the greatest improvement from baseline were anxiety, depression, and appetite (Table 2).

**Table 2.** Changes in Scores for Individual Edmonton Symptom Assessment Scale Items from Days 0 to 14 (*n* = 18)

Variable	Mean change (95% CI)	Median (IQR change)	Range
Pain	−0.61 (−1.78 to 0.56)	−1.0 (−2.0 to 0.0)	−6 to 4
Tiredness	−0.17 (−1.50 to 1.17)	0.0 (−1.0 to 1.0)	−8 to 6
Nausea	−0.56 (−1.91 to 0.79)	−0.5 (−3.0 to 1.0)	−5 to 6
Shortness of breath	−0.5 (−1.59 to 0.59)	0.0 (−2.0 to 0.0)	−4 to 4
Drowsiness	0.22 (−0.92 to 1.37)	0.5 (−1.0 to 1.0)	−4 to 5
Appetite	−0.94 (−1.90 to 0.01)	−1.0 (−2.0 to 1.0)	−4 to 2
Anxiety	−1.61 (−2.92 to −0.30)^*^	−1.0 (−4.0 to 0.0)	−7 to 3
Depression	−1.33 (−2.50 to −0.16)^**^	−1.0 (−2.0 to 0.0)	−8 to 3
Well-being	−0.28 (−1.56 to 1.01)	0.0 (−2.0 to 1.0)	−7 to 4

^*^ *p* = 0.02.

^**^ *p* = 0.03.

IQR, interquartile range.

The median (range) oral morphine equivalent (OME) opioid dose at baseline was 140 mg/day (range 0–800, *n* = 21) and 95 mg/day (0–370, *n* = 18) at day 14. In the 18 participants who completed day 14, there was no change in OME between baseline and day 14 (median [range] 100 mg/day [range 0–420] at baseline and 95 mg/day [range 0–370] at day 14, *p* = 0.09).

### Impression of benefit, anxiety/depression, and QoL

At day 14, 8 of 18 (44.4%) participants reported a PGIC score of 4 or more—reflecting an overall improvement in their condition since starting cannabis. The remainder reported no change or that their condition was worse. The clinician-assessed CGIC at day 14 scored 50% of patients as having had some improvement in their condition, with the remainder having no change or worse.

For the 17 participants who completed the DASS-21 at day 14, the median (range) depression score decreased from 3 (0–11) at baseline to 2 (0–18) at day 14, *p* = 0.04, with the median stress score decreasing from 6 (0–21) at baseline to 3 (0–20) at day 14, *p* = 0.046. The median anxiety score did not have a significant change. The total median DASS scores decreased from 13 (2–40) to 8 (0–50), *p* = 0.047. There was no change in overall QoL as measured by the EORTC.

### Adverse events

The most common AEs reported as worse than baseline at any stage during the study were drowsiness, worsening mood, hypertension, nausea/vomiting, and abdominal pain (Table 3). No AE greater than grade 2 was reported.

**Table 3.** Number of Adverse Events Graded Worse than at Baseline

Adverse event	Days 1–7	Days 8–14	Days 15–21	Days 22–28	Total: CBD and THC
Confusion	2	1	1		4
Somnolence	5	3	2	1	11
Personality change				1	1
Paranoia	1			1	2
Anxiety	2	2		1	5
Mood	1	3		2	6
Psychosis					0
Hypertension	1	3	2		6
Tachycardia	2	1	1		4
Sweating	1	1			2
Nausea	3	4			7
Vomiting	3	2	2		7
Abdominal pain	3	3			6

## Discussion

This pilot study was designed to assess feasibility of using global symptom burden measures to assess response to medicinal cannabis, to determine median tolerated doses of CBD and THC, and to document AEs in preparation for a series of randomized controlled trials of CBD, THC, and their combinations. It was not powered to measure efficacy, with the sample size being dependant on product supplies available.

We confirmed feasibility in that participants were recruited within a short time period, and 86% were able to complete the primary outcome measure at day 14. The mean reduction in TSDS of 5.8 at day 14 in this study is consistent with that shown by Hui et al.^8^ when measuring symptom improvement and suggests that our chosen outcome measure is appropriate. A response rate of just <50% is perhaps less than would have been anticipated in an open label study considering the anticipated placebo effect. An interesting finding was the improvement in emotional ESAS scores. Whether this was a consequence of trial participation and regular follow-up or of medicinal cannabis is unknown and can only be confirmed within the context of a larger placebo-controlled trial.

The majority of patients received the CBD product with a median end dose of 300 mg/day. CBD has been predominately studied in refractory childhood epilepsies using doses of 20 mg/kg.^15^ This is one of the first trials to study higher dose CBD products in advanced cancer patients. The medication was generally well tolerated, the major adverse effect being drowsiness that seemed dose related and improved with a dose reduction. The adverse effect profile is consistent with other studies in highlighting drowsiness and nausea.^4^ In some patients, CBD appeared to lead to a marked improvement in total symptom distress, whereas others had no discernible benefit. The number of patients in this study receiving THC was too small to make any meaningful conclusions.

This study protocol is novel in that it assesses total symptom distress rather than individual symptoms. In a recent study of THC/CBD oromucosal spray medication, although there was no significant difference in pain scores, the patient global impression of change showed a significant difference between groups.^4^ We are hypothesizing that any benefit of medicinal cannabis may be in holistic well-being rather than limited to a single symptom. This study has demonstrated that ESAS TSDS is a feasible and practical measure to assess participant change in symptom distress over time. It has also confirmed the benefit of including emotional well-being subscales.

The major limitations of this study are the small sample size and lack of placebo. The participants were heterogeneous and had complex clinical pictures consistent with their advanced disease and various treatment protocols. Although the median baseline performance status of participants was high (median AKPS 70), the median survival of around five months demonstrates that most patients enrolled had a poor prognosis and clearly fitted the definition of palliative care.

This study has shown that trials of medicinal cannabis in advanced cancer patients undergoing palliative care are feasible. The doses of THC and CBD used in this study were generally well tolerated and the outcome measure of ESAS TSDS is promising as a measure of overall symptom benefit. However, these results need to be replicated within a placebo-controlled trial to test the true effect of medicinal cannabis in this patient population. With the current state of knowledge, we suggest that medicinal cannabis in this patient population should always be prescribed within the context of a clinical trial or through prospective data collection, whereby efficacy and side effects can be monitored in a controlled manner.

## Supplementary Material

Supplemental data
