# Supplementary material for: An Open-Label Pilot Study Testing the Feasibility of Assessing Total Symptom Burden in Trials of Cannabinoid Medications in Palliative Care
Source: J Palliat Med. 2020 May 4;23(5):650–5. doi: 10.1089/jpm.2019.0540 (PMC7232640; doi:10.1089/jpm.2019.0540)
Supplement: Supplemental data [file Supp_Appendix_S1.pdf]

Mater Research

Participant Dosing Schedule Card & Instruction ARM 1 (CBD 100mg/ml) days 0 – 13

Instructions: Days 0 – 13 please take the oil solution as stated below and place a **TICK ☑** in the box to indicate taken. If possible try and take the oil solution at the same time each day. If you miss a dose more than 6hrs late *(except for days 0, 1, 2 and 3,* or vomit, you should skip that dose and start your next dose as per the dosing schedule. You will **NOT** be supplied with extra bottles to make up missed doses. Bring any unused bottles in their original packaging and your completed dosing card to each clinic appointment. Please call the study nurse if unsure about any of the instructions or if you have any questions.

You will be supplied a new bottle at each clinic appointment

Your dosing schedule may vary depending on symptoms and doctor review. Your Doctor will adjust the dosing card to follow the new medication schedule.

* ***Clinic days*** ** ***Phone calls from nurse***

Participant ID: ________________ Start Date: ______________________ End Date: ___________________________

	Day 0*	Day 1	Day 2**	Day 3	Day 4**	Day 5	Day 6
**Date dd/mm/yy**							
Morning – total # of **mls**	0.5 ☐	0.5 ☐	1 ☐	1 ☐	1 ☐	1 ☐	1 ☐
Midday – total # of **mls**							1 ☐
Evening – total # of **mls**					1 ☐	1 ☐	1 ☐
	**Day 7***	**Day 8**	**Day 9****	**Day 10**	**Day 11****	**Day 12**	**Day 13**
**Date dd/mm/yy**							
Morning – total # of **mls**	1 ☐	1 ☐	1 ☐	2 ☐	2 ☐	2 ☐	2 ☐
Midday – total # of **mls**	1 ☐	1 ☐	1 ☐	1 ☐	1 ☐	2 ☐	2 ☐
Evening – total # of **mls**	1 ☐	2 ☐	2 ☐	2 ☐	2 ☐	2 ☐	2 ☐

Study Nurse: ________________________________ Phone #:______________________________________

Mater Research

Participant Dosing Schedule Card & Instruction ARM 1 (CBD100mg/ml) days 14 – 28

Instructions: Days 14 – 28 please take the oil solution as stated below and place a **TICK** ☑ in the box to indicate taken. If possible try and take the oil solution at the same time each day. If you miss a dose more than 6hrs late or vomit, you should skip that dose and start your next dose as per the dosing schedule. You will **NOT** be supplied with extra bottles to make up missed doses. Bring any unused bottles in their original packaging and your completed dosing card to each clinic appointment. Please call the study nurse if unsure about any of the instructions or if you have any questions.

You will be supplied a new bottle each clinic appointment

Your dosing schedule may vary depending on symptoms and doctor review. Your Doctor will adjust the dosing card to follow the new medication schedule.

***Clinic days** ** **Phone calls from nurse** ON DAY 28 return to clinic (no oil solution this day)*

Participant ID: ________________ Start Date: ______________________ End Date: ___________________________

	Day 14*	Day 15	Day 16**	Day 17	Day 18**	Day 19	Day 20
**Date dd/mm/yy**							
Morning – total # of **mls**	2 ☐	2 ☐	2 ☐	2 ☐	2 ☐	2 ☐	2 ☐
Midday – total # of **mls**	2 ☐	2 ☐	2 ☐	2 ☐	2 ☐	2 ☐	2 ☐
Evening – total # of **mls**	2 ☐	2 ☐	2 ☐	2 ☐	2 ☐	2 ☐	2 ☐
	**Day 21***	**Day 22**	**Day 23****	**Day 24**	**Day 25****	**Day 26**	**Day 27**
**Date dd/mm/yy**							
Morning – total # of **mls**	2 ☐	2 ☐	2 ☐	2 ☐	2 ☐	2 ☐	2 ☐
Midday – total # of **mls**	2 ☐	2 ☐	2 ☐	2 ☐	2 ☐	2 ☐	2 ☐
Evening – total # of **mls**	2 ☐	2 ☐	2 ☐	2 ☐	2 ☐	2 ☐	2 ☐

Study Nurse: ________________________________ Phone #:_____________________________________

Mater Research

Participant Dosing Schedule Card & Instruction ARM 2 (THC10mg/ml) days 0 – 13

Instructions: Days 0 – 13 please take the oil solution as stated below and place a **TICK ☑** in the box to indicate taken. If possible try and take the oil solution at the same time each day. If you miss a dose more than 6hrs late *(except for days 0, 1, 2 and 3,* or vomit, you should skip that dose and start your next dose as per the dosing schedule. You will **NOT** be supplied with extra bottles to make up missed doses. Bring any unused bottles in their original packaging and your completed dosing card to each clinic appointment. Please call the study nurse if unsure about any of the instructions or if you have any questions.

You will be supplied a new bottle at each clinic appointment

Your dosing schedule may vary depending on symptoms and doctor review. Your Doctor will adjust the dosing card to follow the new medication schedule.

Clinic days** ** ***Phone calls from nurse

Participant ID: ________________ Start Date: ______________________ End Date: ___________________________

	**Day 0***	**Day 1**	**Day 2****	**Day 3**	**Day 4****	**Day 5**	**Day 6**
**Date dd/mm/yy**							
Morning – total # of **mls**	0.25 ☐	0.25 ☐	0.5 ☐	0.5 ☐	0.5 ☐	0.5 ☐	0.5 ☐
Midday – total # of **mls**							0.5 ☐
Evening – total # of **mls**					0.5 ☐	0.5 ☐	0.5 ☐
	**Day 7***	**Day 8**	**Day 9****	**Day 10**	**Day 11****	**Day 12**	**Day 13**
**Date dd/mm/yy**							
Morning – total # of **mls**	0.5 ☐	0.5 ☐	0.5 ☐	1 ☐	1 ☐	1 ☐	1 ☐
Midday – total # of **mls**	0.5 ☐	0.5 ☐	0.5 ☐	0.5 ☐	0.5 ☐	1 ☐	1 ☐
Evening – total # of **mls**	0.5 ☐	1 ☐	1 ☐	1 ☐	1 ☐	1 ☐	1 ☐

Study Nurse: ________________________________ Phone #:______________________________________

Mater Research

Participant Dosing Schedule Card & Instruction ARM 2 (THC10mg/ml) days 14 – 28

Instructions: Days 14 – 28 please take the oil solution as stated below and place a **TICK ☑** in the box to indicate taken. If possible try and take the oil solution at the same time each day. If you miss a dose more than 6hrs late or vomit, you should skip that dose and start your next dose as per the dosing schedule. You will **NOT** be supplied with extra bottles to make up missed doses. Bring any unused bottles in their original packaging and your completed dosing card to each clinic appointment. Please call the study nurse if unsure about any of the instructions or if you have any questions.

You will be supplied a new bottle each clinic appointment

Your dosing schedule may vary depending on symptoms and doctor review. Your Doctor will adjust the dosing card to follow the new medication schedule.

Clinic days** ** **Phone calls from nurse** **ON DAY 28 return to clinic (no oil solution this day)

Participant ID: ________________ Start Date: ______________________ End Date: ___________________________

	Day 14*	Day 15	Day 16**	Day 17	Day 18**	Day 19	Day 20
**Date dd/mm/yy**							
Morning – total # of **mls**	1 ☐	1 ☐	1 ☐	1 ☐	1 ☐	1 ☐	1 ☐
Midday – total # of **mls**	1 ☐	1 ☐	1 ☐	1 ☐	1 ☐	1 ☐	1 ☐
Evening – total # of **mls**	1 ☐	1 ☐	1 ☐	1 ☐	1 ☐	1 ☐	1 ☐
	**Day 21***	**Day 22**	**Day 23****	**Day 24**	**Day 25****	**Day 26**	**Day 27**
**Date dd/mm/yy**							
Morning – total # of **mls**	1 ☐	1 ☐	1 ☐	1 ☐	1 ☐	1 ☐	1 ☐
Midday – total # of **mls**	1 ☐	1 ☐	1 ☐	1 ☐	1 ☐	1 ☐	1 ☐
Evening – total # of **mls**	1 ☐	1 ☐	1 ☐	1 ☐	1 ☐	1 ☐	1 ☐

Study Nurse: _________________________________ Phone #:_______________________________________
